# Editorial: STEM, STEAM, computational thinking, and coding: Evidence-based research and practice in children's development

**DOI:** 10.3389/fpsyg.2022.1110476

**Published:** 2022-12-13

**Authors:** Stamatios Papadakis, Michail Kalogiannakis, Ali İbrahim Can Gözüm

**Affiliations:** ^1^Faculty of Education, The University of Crete, Crete, Greece; ^2^Dede Korkut Faculty of Education, Kafkas University, Kars, Turkey

**Keywords:** Science Technology Engineering Mathematics (STEM), STEM enriched with arts (STEAM), computational thinking (CT), coding, education

First, we would like to congratulate the research authors and reviewers worldwide for their contributions to the literature with up-to-date scientific information. An overall view of the Research Topics shows that they present up-to-date methodology and results, generating new ideas and alternative perspectives that turn theoretical foundations into practical applications. Another aspect of this particular research compilation is that it contains articles from many different countries, including the United States (USA), Austria, China-Hong Kong, Germany, Greek, Italy, Luxembourg, Malaysia, Palestine, Portugal, Singapore, Taiwan, Turkey and Uruguay. Examination of the frequencies of the keywords used in the 18 different articles in this special issue (see Figure 1) reveals the collection's thematic paradigm.

**Figure 1:**
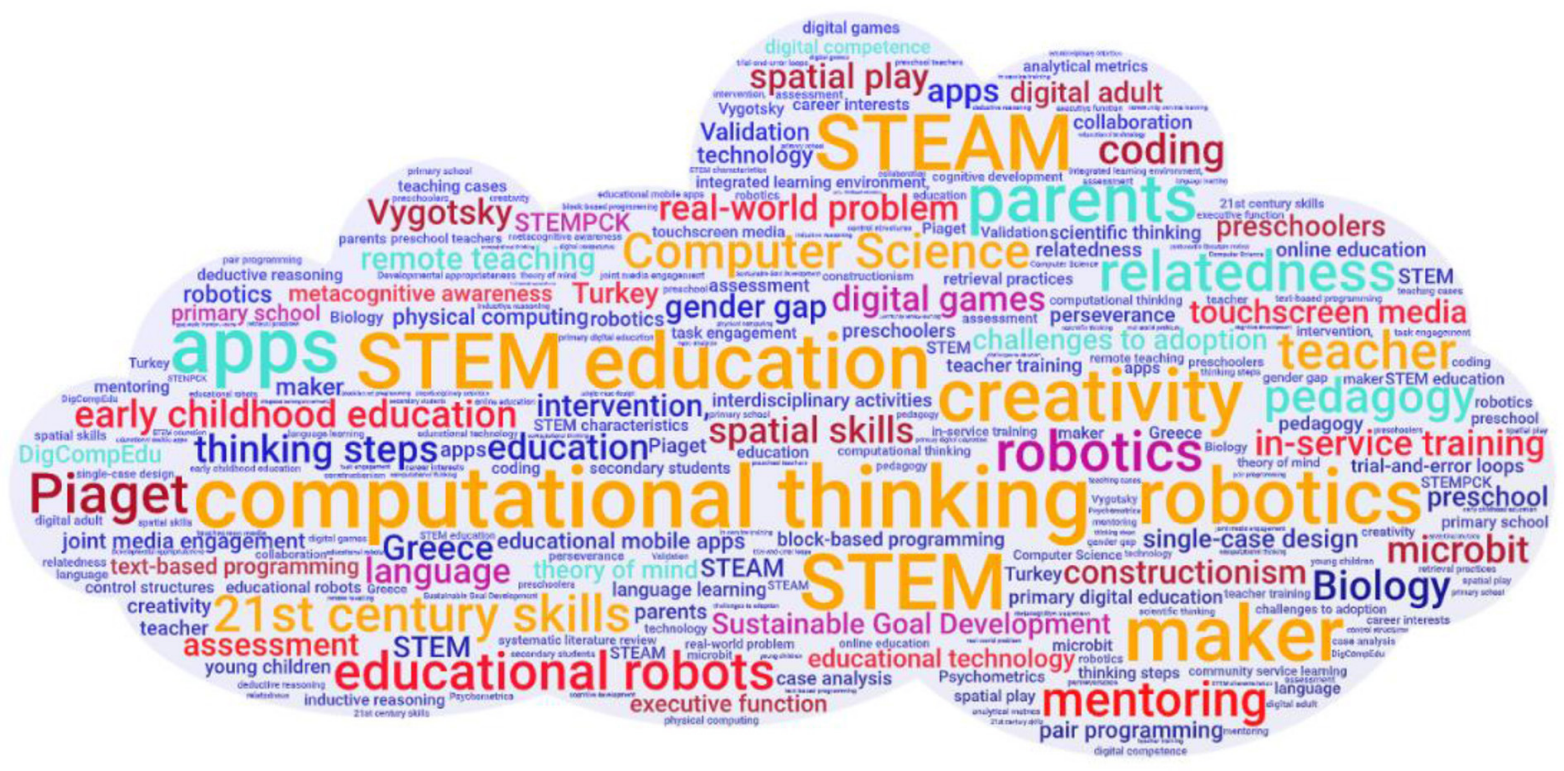
Cloud of research keywords.

It is clear from Figure 1 that the studies frequently use such keywords as Science Technology Engineering Mathematics (STEM), STEM enriched with arts (STEAM), STEM education, computational thinking (CT), robotics, coding, etc. Considering that the title of this special issue is “*STEM, STEAM, computational thinking, and coding: Evidence-based research and practice in children's development,”* we can see that the keywords reflect thematic approaches adopted by the studies. Moving forward, we chose the concept of STEM, one of the main keywords, as the critical keyword that best fits the purpose of the special edition and interpreted its relationship with other concepts. However, we also want to emphasize to our readers the importance of reviewing this special edition regarding the children, parents, and teachers that form the critical triangle in early childhood education, another of the keywords in Figure 1. This being so, it would be helpful to briefly discuss the thematic paradigm of the research concerning children, parents, and teachers.

Regarding child development, STEM education emphasizes cognitive development (computational thinking, creativity, thinking steps, and metacognitive awareness) and twenty-first-century skills for the skills that children today need to have. However, we should keep sight of the fact that STEM activities involving real-world problems, another keyword seen in Figure 1, can support all areas of a child's development. This special edition presents new perspectives to researchers and readers by revealing new findings concerning the relationship between children and parents within the scope of STEM education. What kind of STEM education should children be given? The critical point in this question is the concept of teacher and education. Here, we can see that pedagogical content emerges with critical concepts in both pre-service and in-service teacher training and the teaching of pedagogical content knowledge (PCK) in line with STEM philosophy. As for the concept of teachers, the training programs and technology integration into them (educational robots, robotics, digital games, coding, etc.) also include central key concepts (see Figure 1). This special edition includes 18 research articles and the significant findings of different STEM-oriented research questions asked in these studies.

The first article in this special issue is the study called “*Applying Relatedness to Explain Learning Outcomes of STEM Maker Activities”* conducted by Weng et al.. This study was conducted with the participation of students and teachers in Hong Kong. The researchers examined the effects of STEM maker activities on learning outcomes. They found that maker activities affected the development of learners' non-cognitive characteristics, meaning their cognitive competencies such as STEM interest, STEM identity, and critical thinking by using the relationship between student and mentor and real-world problem (RWP) types.

The second article in this issue is the study *titled “Developing Teaching Practice in Computational Thinking in Palestine”* conducted by Ghani et al.. The researchers aimed to provide information about the challenges faced by CT teachers in K-12 schools in Palestine, the support they provided when incorporating CT strategies in their teaching, and the strategies they adopted when using CT approaches. They found that the most appropriate way to support teachers' CT presentations would be to provide peer exchange and expert coaching concerning the integration of CT into the curriculum.

The third study is an interesting one called “*Children's Spatial Play With a Block Building,”* conducted by Polinsky et al.. The researchers examined the interest of children aged 3–6 years living in the Chicago region of the United States in digital block games and the correlation between children's age, gender, and spatial skills in playing digital block games. Although they found significant differences by age and gender in children playing digital games, they found no correlation between spatial skills and digital games. They reported that physical and digital block games support similar play behavior in children.

Researchers in Luxembourg identified numerous problems in using educational technology in early childhood (STEAM) education. Haas et al. conducted a study called “*Evaluating Technology-Enhanced, Steam-Based Remote Teaching With Parental Support in Luxembourgish Early Childhood Education.”* The researchers collected data from teachers, children, and parents *via* software during the parent-assisted distance learning process to examine the roles of parents in STEAM education and to determine child-parent interaction. They identified new roles in the parent-child relationship in distance STEAM teaching and new opportunities in the use of technology in early childhood education. They also proposed interesting ideas to provide technical knowledge and support for factors that affect children's and parent's perceptions and motivations in distance learning.

The fifth article is the study “*Integrating Computational Thinking and Empowering Metacognitive Awareness in Stem Education”* by Markandan et al.. The study, conducted to support the metacognitive skills of biology students in Malaysia, found significant differences in student achievement by examining their skills at programming the Me-Cot learning module based on four learning theories.

The study *called “How Might We Raise Interest in Robotics, Coding, Artificial Intelligence, STEAM and Sustainable Development in University and On-the-Job Teacher Training?”* by Henze et al. is the sixth article in this issue. The researchers researched the project known as “Qualitätsoffensive Lehrerbildung,” a joint initiative between the Federal Government and the individual States in Germany to improve teacher education quality. The researchers determined the positive effects of the 5E approach on in-class STEM practices based on what pre-service teachers, teachers, and students did on the job according to the 5E approach, the Biological Sciences Curriculum Study, and the pedagogic concept based on the STEM paradigm.

Nikolayev et al.  conducted a single case study with six female participants aged 46–52 months in the USA. The study examined the effect on children's theory-of-mind skills of interactive touch screen apps based on the theory of mind. The study called “*Improving Preschoolers' Theory of Mind Skills With Mobile Games”* found that when children are supported in the use of mobile apps by a conversation they are to have with the help of adults, this can support the development of theory-of-mind skills in preschoolers.

The eighth study, called “*Combined Effects of Block-Based Programming and Physical Computing on Primary Students' Computational Thinking Skills,”* was conducted by Kastner-Haule et al.. By conducting a longitudinal and multi-stage study, the researchers support the increasing importance of computational thinking skills in Austrian classrooms. The study found that block-based programming applications can yield positive results at an early age in support of computing skills.

The ninth article in this issue is the research called “*Exploring Gender Differences in Coding at the Beginning of Primary School,”* conducted by Montuori et al.. Children's coding skills, response blocking, and planning skills were evaluated following the coding training given to first-year students in Italy. The researchers found that the final test results for coding skills following the coding training favored boys. The researchers examined the mediating role of differential executive functions (EF) on gender in coding and found that gender difference had no mediating role effect on EF in coding.

*The study titled “Effects of a Pair Programming Educational Robot-Based Approach on Students' Interdisciplinary Learning of Computational Thinking and Language Learning”* conducted by Hsu et al. is the 10th article in this issue. This research was conducted with the participation of children in Singapore who learned Chinese as a Second Language (CSL) in Singapore and children in Taiwan who learned English as a Foreign Language (EFL). The study's main result was that the children in the EFL group showed better collaborative social skills in Computational Thinking (CT) skills and lower learning anxiety in learning the target language (TL). While the children in the CSL group had better problem-solving skills in CT, they were observed to exhibit more trial-and-error cycle behaviors.

The study called “*Retrieval Practices Enhance Computational and Scientific Thinking Skills,”* conducted by Yaşar et al. is the 11th article in this issue. Researchers conducted class activity research on teaching STEM concepts and CT to secondary school teachers in the USA and how they learned them. The research found that the majority of teachers (96%) understood the retrieval strategies well and how the in-class applications of the related ideas could be tested. The research results can make a significant contribution to the literature in terms of examples of retrieval practices. It is an exciting article that those looking for good practices, especially for developing CT and STE skills, will find helpful.

The study called “*Enhancing Digital Skills of Early Childhood Teachers Through Online Science, Technology, Engineering, Art, Math Training Programs in Estonia,”* conducted by Leoste et al. is the 12th article in this issue. The researchers examined the effects of online activities for early childhood teachers based on STEAM-integrated learning activities. They reported that early childhood educators would increase their digital competencies faster by receiving online training than face-to-face courses.

The 13th article in this issue is the study called “*Promoting Secondary Students' Twenty-First Century Skills and STEM Career Interests Through a Crossover Program of STEM and Community Service Education”* conducted by Huang et al.. After providing 8 weeks of STEM education to secondary school students as part of community service education. The researchers examined the improvement in Hong Kong mass housing residents' problem-solving skills using the information they gained from the real-world problems faced by disadvantaged groups. They observed positive development in the children's creative thinking, cooperation, and STEM career interests.

The study “*Educational Robotics Intervention to Foster Computational Thinking in Preschoolers: Effects of Children's Task Engagement”* by Gerosa et al. makes up the 14th article in this issue. The researchers conducted a quasi-experimental study based on an educational robotics app with children attending a public kindergarten in Uruguay. They examined the improvement in the task commitment, distraction, verbal participation, and goal realization skills of the children in the experimental group using an app called RoboTito. An overall view of the study shows that commitment to task supports children's learning and that robotic intervention supports the development of computational thinking.

The study called “*A Systematic Review of Technologies to Teach Control Structures in Preschool Education”* conducted by Bakala et al. is the 15th article in this issue. The researchers analyzed empirical evidence by examining the tools that constitute those programs that include the control structures of preschool children and by a systematic literature review of the roles played by the tools in question in teaching the control structures of early childhood children. The study is an exciting article for discovering the technologies for teaching children's control structures.

The study called “*Preschool teachers' STEM pedagogical content knowledge: A comparative study of teachers in Greece and Turkey”* by Gözüm et al. is the 16th article in this issue. The researchers compared the STEM pedagogical content knowledge (STEMPCK) levels of Greek and Turkish preschool teachers and determined no significant difference between the STEMPCK scores of Greek and Turkish teachers. The study also revealed the critical importance of STEM education in the STEMPCK differentiation of Greek and Turkish teachers.

The 17th article in this issue is the study called “*K-12 Science, Technology, Engineering, and Math characteristics and recommendations based on analyses of teaching cases in China”* conducted by Zheng et al.. The researchers examine STEM applications in different regions of China from the program's perspective and offer suggestions for practice. They suggest that STEM education in China should be localized for the integrated interdisciplinary application of STEM education and innovative STEM practices to be effective.

The study called “*Comparing the psychometric properties of two primary schools Computational Thinking (CT) assessments for grades 3 and 4: the Beginners' CT test (BCTt) and the competent CT test (cCTt)”* by El-Hamamsy et al. is the 18th and final article in this issue. The researchers compare psychometric characteristics based on the age validity of the data collection tools used to measure children's Computational Thinking skills. They report that the CT test (BCTt) was more beneficial for beginners in identifying children with low abilities.

## Author contributions

All authors listed have made a substantial, direct, and intellectual contribution to the work and approved it for publication.

